# Exploring the impact of light spectra on growth: unveiling the role of LED light in modulating phytochemical responses of feverfew (*Tanacetum parthenium* L*.*)

**DOI:** 10.1186/s12870-026-09411-y

**Published:** 2026-07-02

**Authors:** Razieh Ebrahimi, Keramatollah Saeidi, Masood Ghasemi Ghehsareh, Fatemeh Jamshidi-Kia

**Affiliations:** Department of Horticulture Science, Faculty of Agriculture, Shahrekord University, Shahrekord, Iran

**Keywords:** Antioxidant activity, Light quality, Parthenolide, Photosynthesis, Secondary metabolites, *Tanacetum parthenium*

## Abstract

**Background:**

Light serves as a critical environmental signal regulating plant growth, photosynthesis, and metabolite synthesis through physiological mechanisms. Advances in LED technology enable precise control of light spectra; however, the physiological responses of medicinal plants to different spectral qualities remain insufficiently understood. This study investigated the effects of various LED light spectra on growth performance, photosynthetic characteristics, and phytochemical profiles of *Tanacetum parthenium*.

**Results:**

The experiment was conducted in a controlled greenhouse at Shahrekord University from April 9 to September 6, 2025 (spring–summer growing season) using a completely randomized design. Plants were grown under natural greenhouse photoperiod conditions until the full flowering stage. Supplemental LED treatments (100% red (R), 100% blue (B), 100% white (W), 50% red:50% blue (50R:50B), 50% red:50% white (50R:50W), and natural greenhouse light (control)) were applied at a photosynthetic photon flux density (PPFD) of 100 μmol m⁻^2^ s⁻^1^ for 7 h per night (22:00–05:00 h). The 50R:50B treatment promoted stem elongation (35.15 ± 0.61 cm) and flower number (30.00 ± 2.00), while blue light enhanced flower biomass and chlorophyll a content (2.77 ± 0.07 mg /g FW). Red light significantly increased parthenolide accumulation in leaves (16.73 ± 0.01 mg/g), while the highest flower parthenolide contents were observed under red (31.29 ± 0.01 mg/g) and white (31.25 ± 0.00 mg/g) light treatments. Blue and red–blue spectra elevated total soluble carbohydrates (18.44 ± 0.32 mg /g FW), total phenolic and flavonoid contents, and antioxidant capacity (FRAP: 87.56 ± 0.66 µM Fe^2^⁺/g extract). Mixed spectra increased carotenoid levels. Although numerical differences were observed in chlorophyll fluorescence parameters, no significant differences were detected among treatments (*p* > 0.05), indicating stable PSII performance. Red light resulted in numerically higher PSII efficiency (*Φ*_Po_: 0.81 ± 0.005); electron transport efficiency (*Φ*_Eo_: 0.443 ± 0.002), and white light maximized the performance index (*PI*_Abs_: 977.55 ± 2.88), statistical analysis showed no significant differences among treatments for chlorophyll fluorescence parameters and stable across light treatments.

**Conclusions:**

Supplemental spectral manipulation under greenhouse conditions differentially modulated growth patterns and secondary metabolite accumulation in *T. parthenium* without inducing photochemical stress. These findings highlight the role of wavelength-dependent metabolic regulation in optimizing medicinal plant production.

**Supplementary Information:**

The online version contains supplementary material available at https://doi.org/10.1186/s12870-026-09411-y.

## Introduction

*Tanacetum parthenium* L*.* (Feverfew) is a medicinal plant widely used for its anti-inflammatory and antimigraine properties, primarily due to sesquiterpene lactones such as parthenolide [1–4]. The biosynthesis and accumulation of these metabolites are strongly regulated by environmental factors, with light quality being one of the most influential signals affecting growth, photosynthetic activity, and secondary metabolism [5–7].

Light-emitting diodes (LEDs) have become the preferred technology in controlled-environment agriculture because they provide high energy efficiency, low heat emission, and precise spectral control [8–11]. Previous studies demonstrate that red and blue wavelengths play central roles in regulating plant morphogenesis and enhancing metabolite production; however, plant responses are highly species-specific and dependent on spectral combinations rather than single wavelengths [12–14].

Although LED-based cultivation has been extensively explored in several medicinal plants, research on *T. parthenium* remains limited. Existing studies primarily focus on natural variation in phytochemical composition or traditional cultivation systems [3, 4]. Only a few reports have examined LED effects on feverfew, and these often evaluate a single light quality without comparing monochromatic and mixed spectra [12].

These findings highlight the need for a targeted evaluation of LED spectra on feverfew, particularly considering organ-specific metabolite accumulation. Moreover, organ-specific responses (leaf vs. flower) to different light spectra have not been systematically characterized, despite the known localization of parthenolide in glandular trichomes of floral tissues [15, 16]. Although LEDs have been extensively studied in various medicinal herbs, few studies have comprehensively examined their organ-specific effects on growth, photosynthesis, and metabolite accumulation in *T. parthenium*. In addition, since parthenolide shows different levels in leaves and flowers, the absence of any task-centered studies focused on controlled lighting targeting specific organs represents a significant knowledge gap. Elucidating these organ-specific responses can inform precision cultivation strategies to optimize parthenolide yield and medicinal quality. Therefore, the present study investigates the effects of red, blue, white, and mixed LED spectra on the growth, photosynthetic efficiency, and phytochemical composition of *T. parthenium*. It specifically examines whether spectral composition differentially regulates organ-specific biomass allocation and secondary metabolite accumulation, with particular emphasis on parthenolide and phenolic compounds. In addition, the study evaluates the extent to which spectral manipulation influences carbohydrate metabolism and antioxidant capacity without substantially altering core PSII photochemical efficiency. By addressing these aspects, the research aims to provide insight into precision lighting strategies for improving medicinal quality.

## Materials and methods

### Experimental conditions

This research was executed in controlled environment of Shahrekord University's research greenhouse, situated in the Chaharmahal and Bakhtiari province of Iran (geographical coordinates: 32° 21' N, 50° 49' E; altitude: 2050 m above mean sea level) during the 2025 growing April 9 to September 6, 2025 (spring–summer growing season), The experimental design implemented was a randomized complete design encompassing six replications. Seeds of *T. parthenium* were sourced from Zardband Pharmaceutical Company (National ID 10861684637). The seeds were sown in 72-cell seedling trays containing a 1:1 mixture of cocopeat and perlite and maintained. The greenhouse conditions were maintained at a temperature regime of 22/18 °C (day/night), relative humidity of 60 ± 5%, and an average CO₂ concentration of approximately 360 ppm. The natural midday light intensity at the plant canopy was approximately 250 μmol m⁻^2^ s⁻^1^, due to partial shading provided by partition walls separating the experimental treatments. After four weeks, uniform seedlings were transplanted into plastic pots (20 cm diameter × 20 cm height) filled with a growth medium composed of field soil, perlite, and cocopeat in a 2:1:1 ratio. The plants were then subjected to the designated light treatment regimens.

### Light treatments

**Fig. 1:**
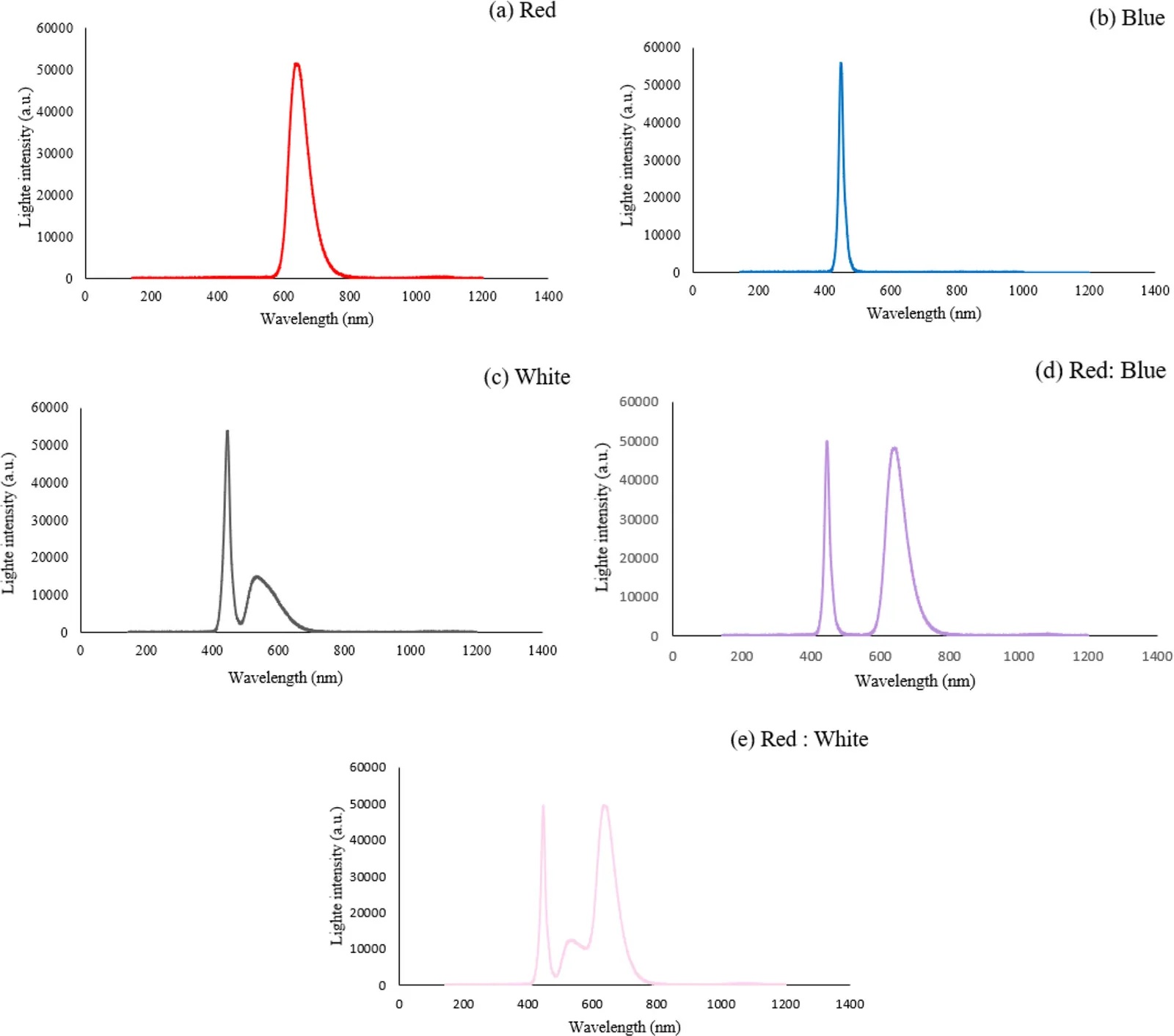
Relative spectral power distribution of the LED light treatments applied during plant growth, including: **a** Red (peak at 630–650 nm), **b** Blue (peak at 460–465 nm), **c** White (3000–3500 K), **d** 50R:50B, and **e** 50R:50W

**Fig. 2:**
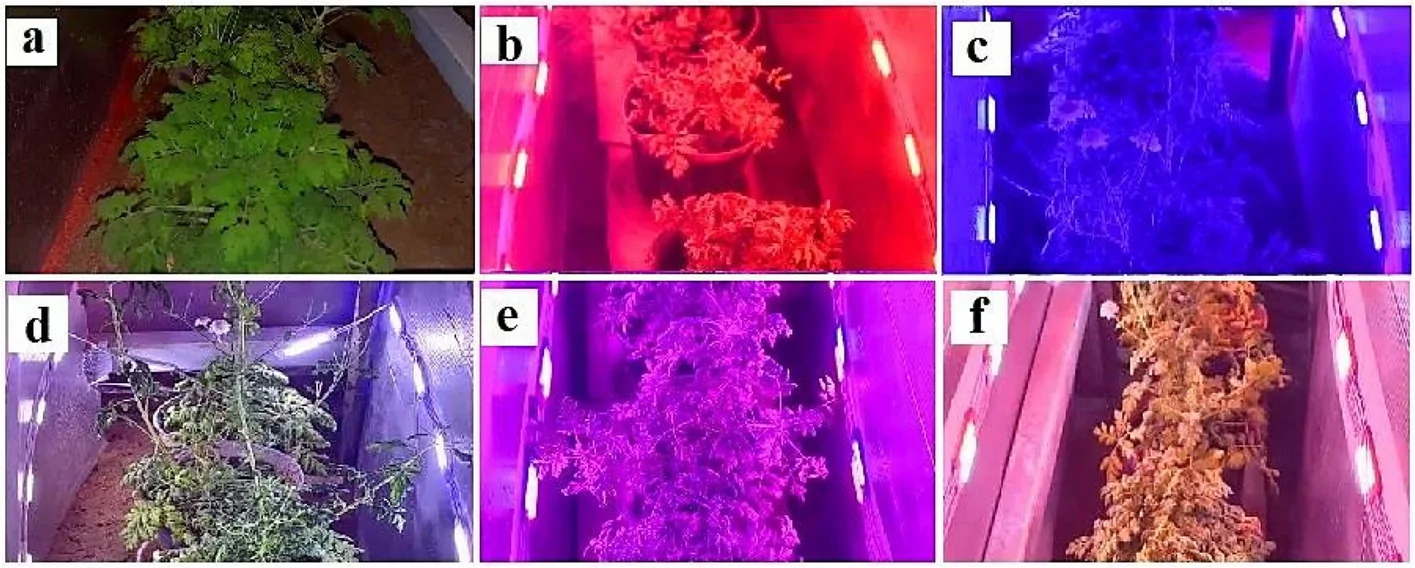
*T. parthenium* under different LED light treatments **a**) Control (C), **b** 100% Red (R), **c** 100% Blue (B), **d** 100% White (W), **e** 50R: 50B%, and **f** 50R:50W

The experiment was conducted in a controlled research greenhouse under natural seasonal photoperiod conditions (approximately 14 h daylight during the growing period). Plants received natural solar radiation during daytime, with a midday photosynthetic photon flux density (PPFD) of approximately 250 μmol m⁻^2^ s⁻^1^ at canopy level. Supplemental LED treatments were applied during the dark period. Light treatments were implemented using 40 units of 2-W, 12-V COB LED modules (Guangdong, China) installed over a cultivation area of 150 × 45 cm (0.675 m2). The LED arrangement was calibrated to provide a uniform photosynthetic photon flux density (PPFD) of 100 μmol m⁻2 s⁻1 at canopy level. The spectral distribution of the applied LED treatments was determined using a V900 spectrometer (Optical Physics Technologists Co., Kashan, Iran) and is presented in Fig. 1. The treatments included 100% Red (R; 630–650 nm), 100% Blue (B; 460–465 nm), 100% White (W; 3000–3500 K), 50R:50B, 50R:50W, and natural greenhouse light served as the control, all adjusted to a photon flux density of 100 μmol m⁻^2^ s⁻^1^ using an LED dimmer and a quantum PAR meter (Apogee MQ-500, USA) (Fig. 2). To prevent light contamination, pots were separated by opaque, reflective plastic sheets installed in an east–west orientation, with LED lamps installed at a distance of 20 cm from the plant canopy Environmental conditions were maintained similar to the greenhouse, except for light quality. Light treatments were applied during the dark period, from 22:00 to sunrise (about 5:00 am) and were supply to 6 pots per treatment. Nighttime spectral treatments were designed to investigate photoreceptor-mediated signaling independently of daytime carbon assimilation. Although photosynthetic activity is limited in the absence of solar radiation, red and blue wavelengths can regulate plant physiology through phytochrome- and cryptochrome-dependent pathways [17]. Red light influences phytochrome dark reversion and circadian regulation, while blue light modulates cryptochrome signaling and downstream gene expression [18]. These pathways are known to affect secondary metabolite biosynthesis, carbohydrate remobilization, and antioxidant regulation beyond direct photosynthetic carbon fixation. Therefore, applying LED treatments during the dark period allowed assessment of wavelength-specific signaling effects without confounding high daytime irradiance responses [19, 20]. Photoperiod length was controlled using automated timers to ensure consistency across treatments. All other environmental conditions including temperature, relative humidity, and irrigation were identical across treatments. To minimize positional effects, pots were systematically rotated every 2–3 days throughout the growth and treatment periods. All morphological, physiological, and biochemical measurements were performed at the full flowering stage, approximately 130 days after planting. This stage was selected to ensure uniform developmental status across treatments and to allow reliable comparison of growth and metabolite responses.

### Growth parameters

Growth parameters, such as plant height, number of flowers, flower diameter, fresh and dry weights of flowers and leaves, and leaf area, were measured at the flowering stage. Plant height was recorded with the help of a standard ruler, whereas flower diameter was recorded with a digital caliper (Model: 0–150 mm, Guanglu Instruments Co., Ltd., Guilin, China) for more accuracy. Fresh weights of floral and foliar tissues were measured with a digital analytical balance (Model: GR-200, A&D Co., Ltd., Tokyo, Japan). Leaf area was measured by analyzing photographs taken under standardized light conditions using Digimizer software (version 4.1.1.0; MedCalc Software, Belgium).

### Chlorophyll fluorescence measurement

Chlorophyll fluorescence parameters were measured at the full flowering stage using a portable PAR-FluorPen (FP 100 max, Photon Systems Instruments, Drásov, Czech Republic). Fully expanded leaves were dark-adapted for 20 min using leaf clips prior to measurement. A saturating light pulse (approximately 3000 μmol m⁻^2^ s⁻^1^, red light) was applied to induce the OJIP fluorescence transient. The maximum quantum yield of PSII (*Φ*_Po_ = *Fv/Fm*), quantum yield of electron transport (*Φ*_Eo_), energy dissipation (*Φ*_Do_), and performance index (*PI*_Abs_) were calculated according to the JIP-test protocol implemented in the FluorPen software. All measurements were performed under identical environmental conditions and instrument settings for all treatments. Fluorescence measurements were conducted using the internal measuring and saturating light source of the instrument; external growth LEDs were not used during measurements, ensuring standardized conditions across treatments.

### Chlorophylls and carotenoids content

Chlorophyll a (Chl *a*), chlorophyll b (Chl *b*), and carotenoid (Car) contents were quantified from mature leaves homogenized in 80% acetone under dark conditions at 25 °C. Absorbance was recorded at 470, 645, and 663 nm using a spectrophotometer (T60 UV–Visible Spectrophotometer, PG Instruments Ltd, Lutterworth, United Kingdom)), and pigment concentrations (mg/g fresh weight (FW)) were calculated using Eq. (1– 3) [21].

1 $$\begin{aligned} \mathrm{C}\mathrm{h}\mathrm{l} \mathrm{a} &= [12.21({\mathrm{A}}_{663}) -2.81({\mathrm{A}}_{646})]\\ &\quad \mathrm{V} / (\mathrm{W} \times 1000) \end{aligned}$$

2 $$\begin{aligned} \mathrm{C}\mathrm{h}\mathrm{l} \mathrm{a} &= [20.13({\mathrm{A}}_{646}) -5.03({\mathrm{A}}_{663})]\\ &\quad \mathrm{V} / (\mathrm{W} \times 1000) \end{aligned}$$

3 $$\begin{aligned} \mathrm{C}\mathrm{a}\mathrm{r} &= \left[(1000\times {\mathrm{A}}_{470}) - (3.27\times \mathrm{C}\mathrm{h}\mathrm{l} a)\right.\\ &\quad \left.- (104\times \mathrm{C}\mathrm{h}\mathrm{l} b)\right] / 229 \end{aligned}$$

where; A: absorbance at the specified wavelengths, V: volume of acetone (mL), and W: fresh weight of the sample (g).

### Determination Total Soluble Carbohydrate Content (TSC)

Total soluble carbohydrate content was quantified spectrophotometrically by the anthrone method, with glucose employed as the calibration standard. Briefly, 0.5 g of plant tissue was extracted with 15 mL 95% ethanol, followed by two re-extractions with 70% ethanol. After centrifugation (PIT320 R Universal, 5000 rpm, 10 min), 100 μL of the combined extract was reacted with 3 mL of anthrone reagent (0.2% in 72% H₂SO₄), heated in a boiling water bath for 9 min, and rapidly cooled. Absorbance was measured at 650 nm (T60 UV–Visible Spectrophotometer, PG Instruments Ltd, Lutterworth, United Kingdom), and results were expressed as mg/g FW [22].

### Determination of antioxidant enzymes activities

#### Preparation leaf enzyme extract

To prepare the leaf enzyme extract, approximately 0.2 g of leaf tissue was rapidly frozen in liquid nitrogen and ground into a fine powder. The powdered tissue was extracted in 2 mL of an ice-cold buffer containing 50 mM potassium phosphate buffer (pH 7.5), 0.1 mM EDTA, 0.1% (V/V) Triton X-100, and 2% (W/V) polyvinylpolypyrrolidone. The homogenate was centrifuged at 10,000 × g for 15 min at 4 °C, and the resulting supernatant was collected for enzyme activity assays.

### Superoxide Dismutase (SOD) Activity

The activity of superoxide dismutase (SOD) was determined by assessing its ability to inhibit the photochemical reduction of nitroblue tetrazolium (NBT). A reaction mixture (2.950 mL) was prepared, containing 50 mM potassium phosphate buffer (pH 7.8), 4 μM riboflavin, 13 mM methionine, 75 μM NBT, 0.1 mM Na-EDTA, and 50 μL of Leaf enzyme extract. The reaction was conducted at 25 °C under fluorescent light (15 W) with an intensity of 50 μmol photons·m⁻^2^·s⁻^1^ for 10 min. The absorbance at 560 nm was recorded using a spectrophotometer [23]. One unit (U) of SOD activity was defined as the amount of enzyme required to inhibit 50% of NBT photoreduction under the assay conditions. The percentage of inhibition was calculated according to Eq. (4):

4 $$\begin{aligned} \mathrm{S}\mathrm{O}\mathrm{D} (\mathrm{U} {\mathrm{g}}^{-1} \mathrm{F}\mathrm{W}) &= (({\mathrm{A}}_{\mathrm{c}}- {\mathrm{A}}_{\mathrm{s}}) \times {\mathrm{V}}_{\mathrm{t}} )/\\ &\quad ({\mathrm{A}}_{\mathrm{c}}\times \mathrm{d}\times {\mathrm{W}}_{\mathrm{S}}\times {\mathrm{V}}_{\mathrm{S}}) \end{aligned}$$

where; A_C_: Absorbance of the control reaction (maximum NBT reduction); A_S_: Absorbance in the presence of the enzyme extract (indicating inhibition by SOD); V_t_: Total volume of the reaction (mL); Path length of the cuvette (cm); Vs: Volume of enzyme extract (mL); Ws: Fresh weight of the plant sample (g) used for extraction.

### Catalase (CAT) Activity

Catalase activity was assessed by monitoring the decomposition of hydrogen peroxide (H₂O₂). The reaction mixture consisted of 3 mL of 50 mM potassium phosphate buffer (pH = 7) and 30 mM H₂O₂. The reaction was initiated by adding 100 μL of the leaf enzyme extract. The rate of H₂O₂ reduction was measured spectrophotometrically by observing the decrease in absorbance at 240 nm for 2 min using a UV–Vis spectrophotometer. The activity was calculated according to Eq. (5):

5 $$\begin{aligned} &\mathrm{C}\mathrm{A}\mathrm{T}(\mathrm{m}\mathrm{m}\mathrm{o}\mathrm{l} {\mathrm{H}}_{2}{\mathrm{O}}_{2} {\mathrm{m}\mathrm{i}\mathrm{n}}^{-1} {\mathrm{g}}^{-1}\mathrm{F}\mathrm{W})\\ & = (({\Delta \mathrm{A}}_{240}/\Delta \mathrm{t})\times {\mathrm{V}}_{\mathrm{t}} )/ (\upvarepsilon \times \mathrm{d}\times {\mathrm{W}}_{\mathrm{S}}\times {\mathrm{V}}_{\mathrm{S}}) \end{aligned}$$

where; ΔA_240_: Decrease in absorbance at 240 nm over the measurement time; Δt: Time of measurement (min); V_t_: Total reaction volume (L); ε: Molar extinction coefficient of H₂O₂ at 240 nm (40 mM⁻^1^·cm⁻^1^); d: Path length of the cuvette (cm); Ws: Fresh weight of the plant sample (g) used for extraction; Vs: Volume of enzyme extract (mL).

#### Guaiacol Peroxidase (GPOD) Activity

Guaiacol Peroxidase activity was determined by monitoring the oxidation of guaiacol in the presence of H₂O₂. The reaction mixture (3 mL) contained 50 mM potassium phosphate buffer (pH 7.0), 1% (v/v) guaiacol, and 0.3% (v/v) H₂O₂. The reaction was initiated by adding 100 μL of the leaf enzyme extract. The oxidation of guaiacol, which results in the formation of a tetraguaiacol chromophore, was monitored spectrophotometrically by measuring the increase in absorbance at 470 nm for 2 min using a UV–Vis spectrophotometer. The activity was calculated according to Eq. (6):

6 $$\begin{aligned} &\mathrm{G}\mathrm{P}\mathrm{O}\mathrm{X} (\mathrm{m}\mathrm{m}\mathrm{o}\mathrm{l} \mathrm{t}\mathrm{e}\mathrm{t}\mathrm{r}\mathrm{a}\mathrm{g}\mathrm{u}\mathrm{a}\mathrm{i}\mathrm{a}\mathrm{c}\mathrm{o}\mathrm{l} {\mathrm{m}\mathrm{i}\mathrm{n}}^{-1} {\mathrm{g}}^{-1}\mathrm{F}\mathrm{W})\\ &=(({\Delta \mathrm{A}}_{470}/\Delta \mathrm{t})\times {\mathrm{V}}_{\mathrm{t}} )/ (\upvarepsilon \times \mathrm{d}\times {\mathrm{W}}_{\mathrm{S}}\times {\mathrm{V}}_{\mathrm{S}}) \end{aligned}$$

where; ΔA470: Change in absorbance at 470 nm over the measurement time; Δt: Time of measurement (min), Vt: Total reaction volume (L); ε: Molar extinction coefficient of tetraguaiacol at 470 nm (26.6 mM⁻^1^·cm⁻^1^); d: Path length of the cuvette (cm); Ws: Fresh weight of the plant sample (g) used for extraction; Vs: Volume of enzyme extract used (mL).

### Determination of bioactive compounds

#### Preparation of extracts

For extract preparation, 10 g of plant material (Leaves and Flowers) was macerated in 100 mL of 60% ethanol and subjected to continuous agitation on a shaker (Model SF1, Cole-Parmer, UK) at ambient temperature for 72 h. The mixture was subsequently filtered using Whatman No. 1 filter paper and concentrated under reduced pressure at 37 ± 2 °C using a rotary evaporator (IKA RV 10, IKA-Werke GmbH & Co. KG, Staufen, Germany). The obtained extract was then desiccated in an incubator (Memmert, Schwabach, Germany) at 37 ± 2 °C.

#### Parthenolide Content (PC)

**Fig. 3:**
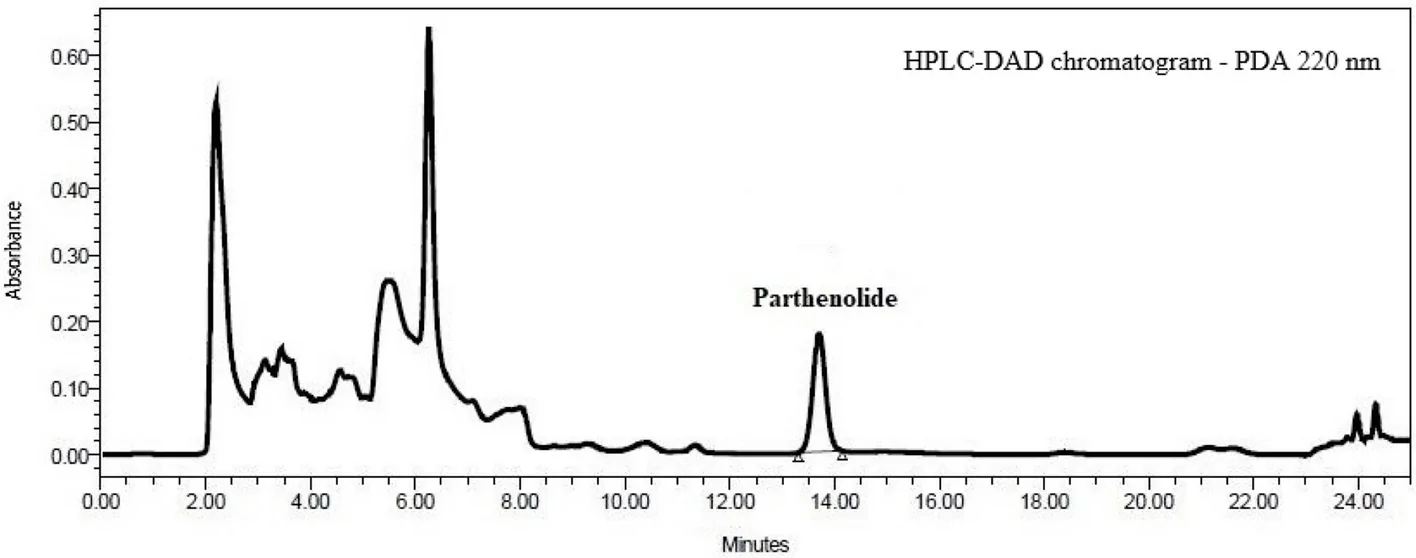
Representative HPLC–DAD chromatogram of the flower extract illustrating the parthenolide peak detected at 220 nm under the 50R:50W light treatment

The Parthenolide Content of extract was quantified by a high-performance liquid chromatography system (Azura, Knauer, Germany) equipped with a Diode Array Detector with detection occurring at 220 nm. Separation was performed on a C18 column (250 × 4.6mm, 5µm). Separation was performed using an isocratic elution with a mobile phase consisting of acetonitrile and water (65:35, v/v) at a flow rate of 1 mL min⁻^1^. The injection volume was 20 µL, the column temperature was maintained at 25 °C. Quantification was carried out based on a calibration curve generated using parthenolide standards (Fig. 3 and Supplementary Data) [24].

#### Determination Total Phenol Content (TPC)

The Total Phenol Content was determined by the Folin–Ciocalteu colorimetric assay using gallic acid as the calibration standard. Briefly, 10 mg dried extract was dissolved in 10 mL of 60% methanol. Subsequently, 200 µL of this solution was mixed with 1 mL of 10% Folin-Ciocalteu reagent and 800 µL of 7.5% sodium carbonate. The mixture was then incubated in darkness for 30 min. Following incubation, absorbance was measured at 765 nm using a UV–Vis spectrophotometer (T60 UV–Visible Spectrophotometer, PG Instruments Ltd, Lutterworth, United Kingdom). TPC was quantified as mg of gallic acid equivalents (GAE) per g of dry extract [25].

#### Determination Total Flavonoid Content (TFC)

The Total Flavonoid Content was quantified using the aluminum chloride colorimetric assay. Briefly, 10 mg of the desiccated extract was reconstituted in 10 mL of 60% methanol. Subsequently, 500 µL of this solution was combined with 500 µL of 2% aluminum chloride and 3 mL of 5% potassium acetate. The resulting mixture underwent a 40 min incubation period in a light-protected environment. Following incubation, the absorbance was determined at 415 nm. Quercetin was the calibration standard, and the TFC was reported as mg of quercetin equivalents (mg QE) per gram of the dry extract [25].

### Antioxidant activity

#### Free radical scavenging activity

The antioxidant activity of the extracts was evaluated using the 2,2-diphenyl-1-picrylhydrazyl (DPPH) radical scavenging assay. Serial dilutions of the extracts in methanol (1 mL) were prepared and subsequently mixed with 2 mL of a 0.1 mM DPPH solution. The resulting mixtures were subjected to a 30 min incubation period in a light-protected environment, following which the absorbance was measured at 517 nm. The IC50 value, representing the concentration of extract required to inhibit 50% of the DPPH radicals, was determined. The inhibition was calculated according to Eq. (7):

7 $$\mathrm{Inhibition}\,({\%})=(({\mathrm{A}_\mathrm{B}}-{\mathrm{A}_\mathrm{A}})/{\mathrm{A}}_{\mathrm{B}}))\times 100$$

where, A_A_ represents the absorbance of the extracts, and A_B_ denotes the absorbance of the control, consisting of 1 mL DPPH solution and 1 mL methanol (without extracts). The blank solution comprised methanol. The IC50 value (mg/mL), defined as the concentration of each sample (mg/mL) required to achieve 50% inhibition of DPPH radical formation, was determined using Microsoft Excel software (Microsoft Excel 2016, KB4011684) (Gulcin & Alwasel, 2023).

### Ferric Reducing Antioxidant Power (FRAP)

**Fig. 4:**
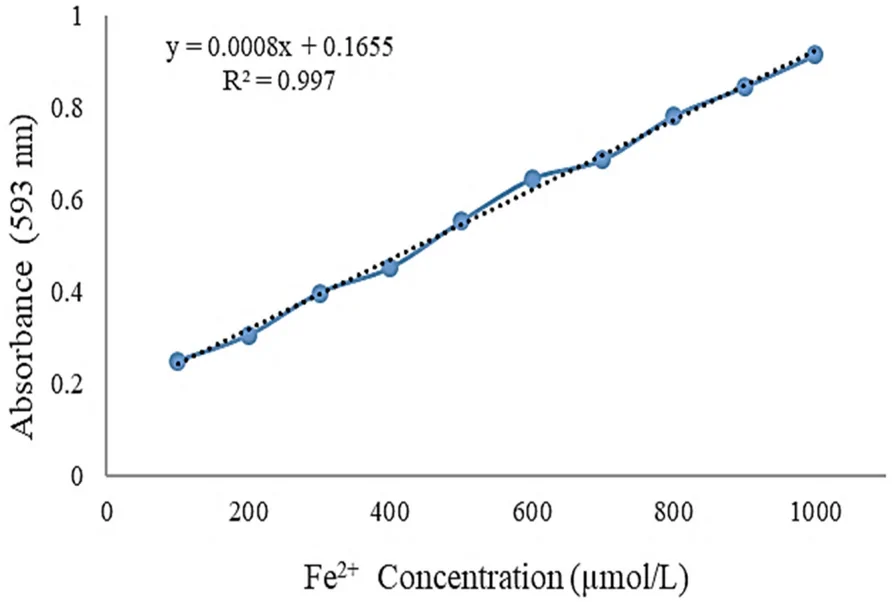
The standard curve of Fe^2+^

The FRAP assay was performed according to the methodology outlined by Benzi and Devaki (2018). The FRAP reagent was prepared immediately prior to use by combining 300 mM sodium acetate trihydrate (in glacial acetic acid, pH 3.6), 10 mM 2,4,6-tri(2-pyridyl)-s-triazine (TPTZ) (in 40 mM HCl), and 20 mM ferric chloride (FeCl_3_) (in deionized water) in a volumetric ratio of 10:1:1 (V/V/V). For each analysis, 3 mL of the FRAP reagent was mixed with 100 μL of the extract’s solution (30 mg/mL in deionized water) and incubated at 37 ± 2 °C for 30 min. The absorbance was then measured at 593 nm using a UV–visible spectrophotometer (LAB INDIA, UV 3000), with a blank solution consisting of 100 μL of deionized water and 3 mL of the FRAP reagent. The FRAP values were determined from a standard curve generated using ferrous sulfate heptahydrate (FeSO_4_·7H_2_O) solutions and expressed as µM Fe^2+^/g extract (Fig. 4) [26].

### Statistical analysis

Statistical analysis was conducted using a completely randomized design (CRD) and one-way analysis of variance (ANOVA) in SPSS version 16.0 (SPSS Inc., USA). Treatment means were subsequently compared using the least significant difference (LSD) test at a significance level of *p* < 0.05. Each treatment consisted of six biological replicates (*n* = 6), and all statistical analyses were performed based on these replicates.

## Results

### Growth parameters

As presented in Table 1, the light spectrum had a significant effect on the morphological traits of *T. parthenium*. Plants grown under the 50R:50B treatment reached the greatest height (35.15 ± 0.61 cm) and produced the highest number of flowers (30.00 ± 2.00), indicating a synergistic effect of red and blue wavelengths on stem elongation and floral development. In contrast, the shortest plants were recorded under 100% white (24.14 ± 0.55 cm) and 100% red (24.27 ± 0.80 cm) treatments. Blue light (100%) significantly enhanced flower fresh weight (6.81 ± 0.95 g), dry weight (1.38 ± 0.21 g), and flower diameter (16.98 ± 0.88 mm), suggesting its critical role in biomass accumulation and flower enlargement. Regarding leaf traits, white light and control conditions promoted the largest leaf areas (23.28 ± 0.37 and 23.71 ± 0.60 cm^2^, respectively), whereas 100% blue markedly reduced leaf expansion (12.70 ± 0.05 cm^2^). Interestingly, the 50R:50W treatment resulted in the highest leaf fresh (40.48 ± 0.64 g) and dry weights (9.34 ± 0.51 g), highlighting its efficiency in vegetative biomass production. Overall, 50R:50B treatment optimized reproductive growth, while 50R:50W promoted vegetative biomass accumulation.

**Table 1:** Effect of different light spectra on growth parameters of *T. parthenium*

	Treatment					
Parameters	100% White (W)	100% Red (R)	100% Blue (B)	50R:50B%	50R:50W%	Control
Plant height (cm)	24.14 ± 0.55^e^	24.27 ± 0.80^e^	28.08 ± 0.37^c^	35.15 ± 0.61^a^	26.81 ± 0.55^d^	29.50 ± 0.00^b^
Flower No	19.67 ± 0.58^c^	26.67 ± 1.53^b^	28.00 ± 2.65^ab^	30.00 ± 2.00^a^	19.00 ± 1.00^c^	18.00 ± 0.00^c^
Flower D. (mm)	16.65 ± 0.92^ab^	16.10 ± 0.70^ab^	16.98 ± 0.88^a^	16.98 ± 0.46^ab^	15.32 ± 0.66^b^	17.70 ± 0.02^ab^
Flower FW (g)	1.95 ± 0.85^d^	4.34 ± 0.55^bc^	6.81 ± 0.95^a^	5.49 ± 0.16^b^	2.53 ± 0.89^d^	3.73 ± 0.00^ cd^
Flower DW (g)	0.30 ± 0.38^b^	1.10 ± 0.25^a^	1.38 ± 0.21^a^	1.42 ± 0.10^a^	0.45 ± 0.20^b^	0.52 ± 0.01^b^
Leaf area (cm^2^)	23.28 ± 0.37^a^	17.85 ± 0.83^b^	12.70 ± 0.05^d^	13.83 ± 0.10^c^	14.46 ± 0.21^c^	23.71 ± 0.60^a^
Leaf FW (g)	20.97 ± 0.14^d^	29.55 ± 0.30^c^	31.39 ± 0.36^b^	31.21 ± 1.27^bc^	40.48 ± 0.64^a^	30.30 ± 1.62^bc^
Leaf DW (g)	4.96 ± 0.03^d^	6.67 ± 0.54^c^	7.39 ± 0.14^b^	7.41 ± 0.36^b^	9.34 ± 0.51^a^	7.16 ± 0.38^bc^

Values are expressed as means ± standard deviation (*n* = 6)

Values are expressed as means ± standard deviation. Mean values within each row followed by the same letters were not significantly at the 5% level different according to the LSD test (*P* < 0.05)

*No* Number, *D* Diameter, *FW* Fresh weight, *DW* Dry weight

### Chlorophyll fluorescence

The one-way ANOVA indicated that none of the chlorophyll fluorescence parameters differed significantly among treatments (*p* > 0.05). However, Numerical variations in chlorophyll fluorescence parameters were observed among treatments (Table 2). The highest PSII efficiency (*Φ*_Po_) was recorded under 100% red light (0.810 ± 0.001), followed by 100% blue (0.797 ± 0.001) and 50R:50B (0.791 ± 0.013), whereas the lowest value was observed under 50R:50W (0.780 ± 0.022). Similarly, *Φ*_Eo_ values ranged from 0.443 ± 0.021 under red light to 0.342 ± 0.074 under 50R:50W. The lowest *Φ*_Do_ (0.189 ± 0.003) was observed under red light, while slightly higher dissipation values were recorded under blue and 50R:50W treatments. The performance index (*PI*_Abs_) was numerically highest under 100% white light (977.55 ± 2.881) and lowest under the control treatment (942.91 ± 4.113).

**Table 2:** Effect of different light spectra on chlorophyll fluorescence parameters of *T. parthenium*

	Chlorophyll fluorescence parameters			
Treatment	*Φ*_*Po*_	*Φ*_Eo_	*Φ*_Do_	*PI*_Abs_
100% White (W)	0.789 ± 0.014^ ns^	0.388 ± 0.018^ ns^	0.211 ± 0.011^ ns^	977.55 ± 2.881^ ns^
100% Red (R)	0.81 ± 0.001^ ns^	0.443 ± 0.021^ ns^	0.189 ± 0.003^ ns^	964.03 ± 8.242^ ns^
100% Blue (B)	0.797 ± 0.003^ ns^	0.426 ± 0.021^ ns^	0.203 ± 0.001^ ns^	942.91 ± 4.113^ ns^
50R:50B%	0.791 ± 0.013^ ns^	0.383 ± 0.033^ ns^	0.209 ± 0.024^ ns^	972.2 ± 7.541^ ns^
50R:50W%	0.78 ± 0.022^ ns^	0.342 ± 0.074^ ns^	0.22 ± 0.024^ ns^	973.78 ± 5.281^ ns^
Control	0.779 ± 0.012^ ns^	0.382 ± 0.023^ ns^	0.221 ± 0.022^ ns^	974.81 ± 3.492^ ns^

Values are expressed as means ± standard deviation (*n* = 6)

*Φ*_Po_: Maximum quantum yield of PSII; *Φ*_Eo_: Efficiency of electron transport; *Φ*_Do_: Quantum yield of energy dissipation; *PI*_Abs_: Performance index on absorption basis. ns: not significantly different at the 5% level according to the LSD test (*P* < 0.05)

### Chlorophylls and carotenoids content

Light quality significantly influenced pigment content in *T. parthenium*, including chlorophyll a (Chl *a*), chlorophyll b (Chl *b*), and carotenoids (P < 0.05) (Table 3). The highest Chl a concentration was observed under 100% blue light (2.77 ± 0.07 mg/g FW), significantly exceeding all other treatments. This was followed by 100% white light (2.37 ± 0.12 mg/g FW), whereas the lowest Chl a content occurred under 100% red light (1.08 ± 0.21 mg/g FW). Regarding Chl b, the highest values were recorded under 100% blue (0.62 ± 0.02 mg/g FW) and 50R:50W% (0.62 ± 0.06 mg/g FW) treatments, which were statistically similar. These were followed by Control (0.57 ± 0.03 mg/g FW), 50R:50B% (0.52 ± 0.04 mg/g FW), and 100% white (0.50 ± 0.07 mg/g FW), with the lowest Chl b under 100% red light (0.45 ± 0.08 mg/g FW).

**Table 3:** Effect of different light spectra on chlorophylls and carotenoids content of *T. parthenium*

Treatment	Chl *a* (mg/g FW)	Chl *b* (mg/g FW)	Carotenoids (mg/g FW)
100% White (W)	2.37 ± 0.12^b^	0.50 ± 0.07^b^	2.64 ± 0.25^bc^
100% Red (R)	1.08 ± 0.21^d^	0.45 ± 0.08^c^	2.59 ± 0.17^bc^
100% Blue (B)	2.77 ± 0.07^a^	0.62 ± 0.02^a^	2.38 ± 0.04^c^
50R:50B%	1.70 ± 0.23^c^	0.52 ± 0.04^ab^	3.11 ± 0.10^a^
50R:50W%	1.66 ± 0.23^c^	0.62 ± 0.06^a^	3.15 ± 0.15^a^
Control	1.74 ± 0.19^c^	0.57 ± 0.03^ab^	2.87 ± 0.16^ab^

Values are expressed as means ± standard deviation (*n* = 6). Mean values within columns followed by the same letters were not significantly different at the 5% level according to the LSD test (*P* < 0.05)

For carotenoids, mixed light treatments (50R:50W% and 50R:50B%) significantly enhanced accumulation, reaching 3.15 ± 0.15 and 3.11 ± 0.10 mg/g FW, respectively. The lowest carotenoid content was observed under 100% blue light (2.38 ± 0.04 mg/g FW), while 100% white (2.64 ± 0.25 mg/g FW), 100% red (2.59 ± 0.17 mg/g FW), and Control (2.87 ± 0.16 mg/g FW) exhibited intermediate levels.

### Total Soluble Carbohydrate Content (TSC)

The results revealed that light quality significantly influenced the total soluble carbohydrate content in *T. parthenium* leaves (*P* < 0.05) (Table 4). The highest accumulation occurred under 100% blue light (18.44 ± 0.32 mg/g FW), followed by 50R:50B% (17.88 ± 0.00 mg/g FW). The lowest carbohydrate contents were observed in the Control (14.74 ± 0.56 mg/g FW) and 100% white light (14.86 ± 0.16 mg/g FW) treatments, with no significant difference between them. These findings suggest that monochromatic blue light and the synergistic combination of red and blue light effectively enhanced carbohydrate biosynthesis [27, 28]. The superior performance under blue and red–blue light spectra can be attributed to enhanced photosynthetic activity and increased carbohydrate accumulation [29, 30].

**Table 4:** Effect of different light spectra on total soluble carbohydrate content of *T. parthenium*

Treatment	Total soluble carbohydrate (mg/g FW)
100% White (W)	14.86 ± 0.16^e^
100% Red (R)	15.31 ± 0.22^de^
100% Blue (B)	18.44 ± 0.32^a^
50R:50B%	17.88 ± 0.00^b^
50R:50W%	16.92 ± 0.11^c^
Control	14.74 ± 0.56^e^

Values are expressed as means ± standard deviation (*n* = 6). Mean values within columns followed by the same letters were not significantly different at the 5% level according to the LSD test (*P* < 0.05)

### Antioxidant enzymes activities

The activities of the antioxidant enzymes CAT, GPOX, and SOD varied significantly under different light treatments (*P* < 0.05) (Table 5). CAT activity was highest in the Control (0.70 mmol H₂O₂ min⁻^1^ g⁻^1^ FW) and declined significantly under 100% red and 100% white light treatments. The 50R:50B% combination maintained relatively high CAT activity, consistent with reports showing that mixed light conditions sustain basal catalase levels for efficient H₂O₂ detoxification [31, 32]. SOD activity followed a different pattern, with the highest value recorded under Control (0.59 U g⁻^1^ FW), followed by 50R:50B% (0.47 U g⁻^1^ FW), whereas 100% red and 100% white light significantly decreased SOD activity. GPOX activity was highest in the Control (0.29 mmol tetraguaiacol min⁻^1^ g⁻^1^ FW) and significantly reduced under 100% red and 50R:50B% treatments. In contrast, 100% blue light maintained relatively high GPOX activity compared to 100% red and 50R:50B%.

**Table 5:** Effect of different light spectra on antioxidant enzymes activities of *T. parthenium*

Treatment	CAT (mmol H2O2 min ^−1^ g ^−1^ FW)	SOD (U g ^−1^ FW)	GPOX (mmol tetraguaiacol min ^−1^ g ^−1^ FW)
100% White (W)	0.55 ± 0.01^c^	0.044 ± 0.00^c^	0.087 ± 0.01^d^
100% Red (R)	0.49 ± 0.02^d^	0.17 ± 0.01^b^	0.053 ± 0.01^e^
100% Blue (B)	0.59 ± 0.02^bc^	0.20 ± 0.03^b^	0.16 ± 0.02^b^
50R:50B%	0.64 ± 0.05^b^	0.47 ± 0.04^a^	0.043 ± 0.02^e^
50R:50W%	0.56 ± 0.04^c^	0.22 ± 0.07^b^	0.14 ± 0.02^c^
Control	0.70 ± 0.04^a^	0.59 ± 0.01^a^	0.29 ± 0.02^a^

Values are expressed as means ± standard deviation (*n* = 6). Mean values within columns followed by the same letters were not significantly different at the 5% level according to the LSD test (*P* < 0.05)

### Parthenolide content

Parthenolide content (PC) varied significantly among light treatments in both leaves and flowers of *T. parthenium* (*P* < 0.05) (Table 6). In leaves, the highest PC was observed under 100% red light (16.73 ± 0.01 mg/g extract), followed by 100% blue light (13.62 ± 0.01 mg/g extract) and the 50R:50B% treatment (11.51 ± 0.02 mg/g extract). The lowest leaf PC was recorded in the Control (7.60 ± 0.00 mg/g extract), significantly lower than all other treatments.

**Table 6:** Effect of different light spectra on parthenolide content of *T. parthenium*

	PC of extract (mg/g extract)	
Treatment	Leaves	Flowers
100% White (W)	9.90 ± 0.022^d^	31.25 ± 0.003^a^
100% Red (R)	16.73 ± 0.010^a^	31.29 ± 0.011^a^
100% Blue (B)	13.62 ± 0.011^b^	28.32 ± 0.020^b^
50R:50B%	11.51 ± 0.023^c^	22.46 ± 0.019^d^
50R:50W%	9.72 ± 0.010^e^	26.05 ± 0.013^c^
Control	7.60 ± 0.002^f^	19.30 ± 0.023^e^

Values are expressed as means ± standard deviation (*n* = 6). Mean values within columns followed by the same letters were not significantly different at the 5% level according to the LSD test (*P* < 0.05)

In flowers, 100% red light resulted in the highest parthenolide content (31.29 ± 0.01 mg/g extract), followed closely by 100% white light (31.25 ± 0.00 mg/g extract). However, no statistically significant difference was observed between these two treatments (*p* > 0.05), indicating that both light spectra were similarly effective in promoting parthenolide accumulation in flowers. Lower parthenolide levels were observed under 100% blue light (28.32 ± 0.02 mg/g extract) and 50R:50W% (26.05 ± 0.01 mg/g extract), while the lowest flower PC occurred in 50R:50B% (22.46 ± 0.02 mg/g extract) and Control (19.30 ± 0.02 mg/g extract).

### Total phenol and flavonoid content

Both total phenolic content (TPC) (Table 7) and total flavonoid content (TFC) (Table 8) varied significantly among light treatments and plant organs (*P* < 0.05). In leaves, the Control exhibited the highest TPC (128.51 ± 0.16 mg GAE/g extract) and TFC (44.51 ± 0.33 mg QE/g extract), whereas the lowest values were recorded under 50R:50W% light (TPC: 49.85 ± 0.74 mg GAE/g extract; TFC: 29.44 ± 0.13 mg QE/g extract). In flowers, the highest TPC was observed under 100% white light (157.55 ± 0.42 mg GAE/g extract), followed closely by 50R:50B% (151.80 ± 0.16 mg GAE/g extract), while the lowest occurred in the Control (67.78 ± 0.47 mg GAE/g extract). For TFC, the 50R:50B% treatment produced the highest content (53.37 ± 0.25 mg QE/g extract), whereas the Control had the lowest (35.77 ± 0.22 mg QE/g extract).

**Table 7:** Effect of different light spectra on total phenol content of *T. parthenium*

	TPC of extract (mg GAE/g extract)	
Treatment	Leaves	Flowers
100% White (W)	109.24 ± 0.32^b^	157.55 ± 0.42^a^
100% Red (R)	94.63 ± 0.55^d^	122.67 ± 0.32^e^
100% Blue (B)	72.35 ± 0.32^e^	145.86 ± 0.82^c^
50R:50B%	99.38 ± 0.16^c^	151.80 ± 0.16^b^
50R:50W%	49.85 ± 0.74^f^	126.32 ± 0.42^d^
Control	128.51 ± 0.16^a^	67.78 ± 0.47^f^

Values are expressed as means ± standard deviation (*n* = 6). Mean values within columns followed by the same letters were not significantly different at the 5% level according to the LSD test (*P* < 0.05)

**Table 8:** Effect of different light spectra on total flavonoid content of *T. parthenium*

	TFC of extract (mg QE/g extract)	
Treatment	Leaves	Flowers
100% White (W)	34.63 ± 0.25^d^	51.17 ± 0.26^b^
100% Red (R)	38.22 ± 0.44^b^	47.38 ± 0.19^d^
100% Blue (B)	37.33 ± 0.26^c^	45.43 ± 0.41^e^
50R:50B%	31.93 ± 0.26^e^	53.37 ± 0.25^a^
50R:50W%	29.44 ± 0.13^f^	48.18 ± 0.25^c^
Control	44.51 ± 0.33^a^	35.77 ± 0.22^f^

Values are expressed as means ± standard deviation (*n* = 6). Mean values within columns followed by the same letters were not significantly different at the 5% level according to the LSD test (*P* < 0.05)

### Antioxidant activity

**Fig. 5:**
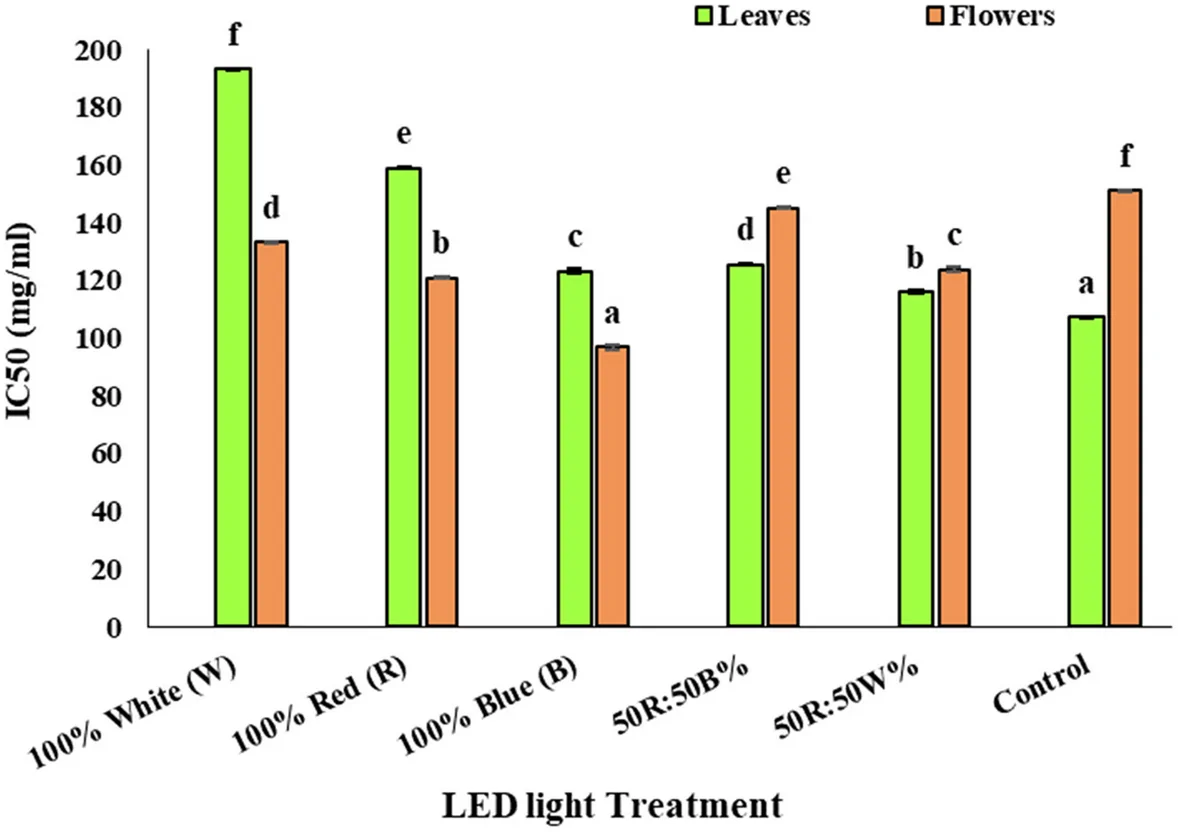
Effect of different light spectra on antioxidant activity (DPPH) of *T. parthenium.* Mean values within columns followed by the same letters were not significantly different at the 5% level according to the LSD test (*P* < 0.05)

**Fig. 6:**
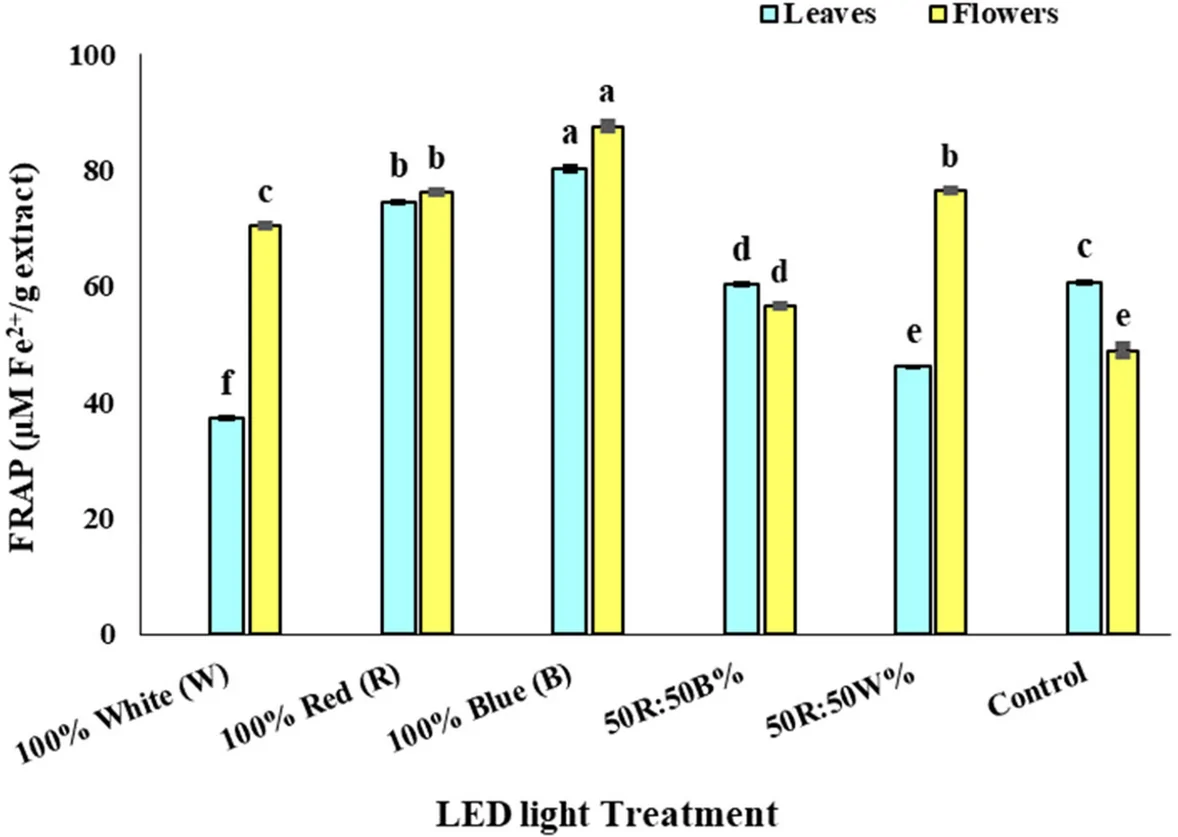
Effect of different light spectra on antioxidant activity (FRAP) of *T. parthenium.* Mean values within columns followed by the same letters were not significantly different at the 5% level according to the LSD test (*P* < 0.05)

Antioxidant activity, as assessed by both DPPH (Fig. 5) and FRAP assays (Fig. 6), showed significant variation across light treatments and between plant organs (*P* < 0.05). In leaf tissues, the lowest DPPH value indicating the highest radical scavenging activity was recorded under the control (107.38 ± 0.10 µg/mL), while the highest DPPH value (193.22 ± 0.01 µg/mL) was observed under 100% white light, reflecting the weakest antioxidant response. In contrast, FRAP values revealed a different trend, with the highest reducing power detected under 100% blue light (80.27 ± 0.62 µM Fe^2+^/g extract), significantly higher than the control and other treatments. In flower tissues, a reverse pattern was observed in the DPPH assay: the highest DPPH value (151.06 ± 0.28 µg/mL) was obtained under the control, while 100% blue light resulted in the lowest value (96.82 ± 0.64 µg/mL), indicating stronger antioxidant potential. Similarly, the maximum FRAP value in flowers was recorded under 100% blue light (87.56 ± 0.66 µM Fe^2+^/g extract), whereas the lowest was found in the control group (48.90 ± 0.92 µM Fe^2+^/g extract).

## Discussion

These results demonstrate that spectral composition distinctly modulates vegetative and reproductive growth allocation in *T. parthenium* under controlled conditions. The observed differences in growth parameters indicate distinct physiological responses of *T. parthenium* to spectral composition. The red–blue combination significantly enhanced stem elongation and floral induction, likely due to the complementary roles of red light in elongation-related responses and blue light in improving photosynthetic efficiency [33, 34]. These responses are consistent with previous reports of wavelength-dependent regulation of plant morphology [35, 36]. Blue light enhanced floral biomass, probably by activating enzymes involved in carbon fixation and flowering, while simultaneously reducing leaf area, reflecting a physiological shift toward reproductive growth [37–39].

The absence of significant differences in chlorophyll fluorescence parameters suggests that the applied light spectra did not induce photoinhibitory stress in *T. parthenium*. Recent studies have emphasized that stable chlorophyll fluorescence parameters generally indicate preserved PSII functionality under moderate light manipulation. Moreover, studies on spectral LED environments have shown that different light qualities can trigger species-specific responses in growth, secondary metabolites, and chlorophyll fluorescence without necessarily altering core PSII efficiency [34, 40–42]. Recent reviews further emphasize that manipulation of blue and red wavelengths can orchestrate metabolic pathways related to secondary metabolite biosynthesis through photoreceptor-mediated signaling [43]. These findings support the idea that the spectral regulation observed in the present study likely reflects downstream metabolic regulation rather than photochemical impairment [34, 38, 44, 45].

The observed enhancement of pigment accumulation under blue and white light is likely associated with improved photosynthetic activity induced by specific wavelengths. Blue light plays a crucial role in chloroplast development and chlorophyll accumulation, consistent with its well-documented regulatory role in photosynthetic processes [46–48]. Consistently, studies report that white or blue light promotes higher chlorophyll and carotenoid production, with blue light specifically enhancing β-carotene and violaxanthin biosynthesis. In contrast, sole red light exposure leads to lower pigment levels [49–52].

The increased carotenoid content under combined red–blue and red–white light treatments indicates a synergistic interaction among multiple photoreceptors, potentially enhancing photoprotection and antioxidant activity [53, 54]. In the present study, such synergistic effects were reflected by significantly higher carotenoid levels under combined light treatments compared with monochromatic spectra. This synergistic effect likely enhanced photoprotection and photosynthetic stability under mixed spectra under combined light spectra. These findings are in agreement with previous reports indicating that red–blue combinations may be more effective than single-wavelength lighting in certain species [55, 56]. Conversely, the reduced Chl *a* and Chl *b* under sole red light exposure leads to lower pigment levels, likely due to the absence of complementary blue-light-dependent regulatory responses [57–59].

The significant increase in total soluble carbohydrates under blue and red–blue light indicates that spectral quality influenced carbon assimilation and allocation in *T. parthenium*. Although LED treatments were applied during nighttime, plants received full natural solar radiation during the day, suggesting that carbohydrate accumulation was not solely attributable to increased daytime photosynthesis but may involve wavelength-dependent metabolic regulation [43, 60]. Blue-rich spectra may have modulated carbon metabolism through photoreceptor- and circadian-mediated signaling pathways [34]. Beyond serving as energy reserves, soluble carbohydrates function as signaling molecules regulating growth and secondary metabolite biosynthesis. Therefore, the elevated TSC under blue and red–blue light likely reflects enhanced carbon availability for downstream pathways, including phenolic and terpenoid synthesis, indicating a shift in metabolic allocation rather than direct changes in PSII efficiency [61–63].

The activities of the antioxidant enzymes CAT, GPOX, and SOD varied significantly under different light treatments and indicating that light quality modulates the plant’s oxidative defense mechanisms. CAT activity was highest in the control treatment. Previous studies suggest that regular light–dark cycles enhance catalase activity, likely because dark periods protect the enzyme from light-induced degradation [64]. The elevated SOD under 50R:50B% suggests that this light combination may stimulate superoxide production, suggesting a potential improvement in ROS-scavenging balance under this light combination [65, 66]. Previous studies indicate that blue light specifically enhances peroxidase activity, contributing to the overall activation of the plant’s antioxidant defense to protect against oxidative stress [67, 68]. Collectively, these enzyme responses indicate that spectral quality does not uniformly enhance antioxidant activity but instead redistributes ROS-scavenging balance depending on wavelength-specific metabolic demands [56, 69–71].

Across all light treatments, parthenolide accumulation was consistently higher in flowers than in leaves, likely reflecting the preferential localization of sesquiterpene lactones in glandular trichomes predominantly present on inflorescences [72–75]. Light quality influenced parthenolide accumulation, with the highest values being observed under red and white light treatments. These findings are consistent with previous studies showing that red wavelengths, perceived through phytochrome signaling, and broad-spectrum white light can regulate secondary metabolism in medicinal plants [34, 76]. The elevated parthenolide levels observed under red and white light treatments suggest that these spectra may promote sesquiterpene lactone accumulation, although the underlying regulatory mechanisms require further molecular investigation [77]. Additionally, 100% blue light increased leaf PC (13.62 mg/g extract), albeit less than red light, consistent with reports that Blue light may modulate secondary metabolite accumulation through redox-related and oxidative signaling processes [34, 78–80]. The observed parthenolide accumulation under red light may be associated with phytochrome-mediated modulation of the cytosolic mevalonate (MVA) pathway, although transcriptomic or enzymatic validation would be required to confirm this hypothesis [34, 43, 81]. In parallel, the increased soluble carbohydrate levels observed under blue and mixed spectra suggest enhanced substrate availability and sugar-mediated signaling, both of which are known to regulate secondary metabolism [56]. Since chlorophyll fluorescence parameters remained stable, the observed variation in parthenolide accumulation appears to be linked more to wavelength-dependent metabolic regulation than to direct changes in PSII photochemical efficiency [43]. The consistently higher parthenolide levels in flowers further indicate tissue-specific carbon allocation toward sesquiterpene biosynthesis, likely reflecting the stronger metabolic sink strength of reproductive tissues [82–85].

These findings are consistent with previous reports by Li et al. (2021), who demonstrated that red and blue light combinations could stimulate flavonoid biosynthesis in reproductive tissues of medicinal plants [86]. Similarly, previous studies have reported that white light can enhance phenolic compound production [87, 88]. Such organ-specific responses highlight the complex interplay between light spectra and plant developmental stages, emphasizing the importance of tailored light strategies in controlled environment agriculture [13, 89]. These responses may be linked to coordinated light-quality–dependent regulation of flavonoid biosynthetic pathways in reproductive tissues. Variations in photoreceptor signaling under different spectral compositions could influence phenylpropanoid metabolism and tissue-specific accumulation patterns. Such organ-specific differences likely reflect the interaction between developmental stage, glandular trichome density, and spectral regulation of secondary metabolism [77, 90].

The observed variation in antioxidant activity among light treatments was closely linked to the accumulation of secondary metabolites, particularly parthenolide, TPC, and TFC. Leaves and flowers with higher parthenolide content exhibited enhanced radical scavenging activity, suggesting a potential contribution of sesquiterpene lactones to antioxidant capacity. Similarly, treatments that promoted TPC and TFC accumulation, such as 50R:50B% and 100% white light in flowers, corresponded with higher FRAP values, highlighting a strong correlation between phenolic/flavonoid content and reducing power. These findings are consistent with previous studies showing that secondary metabolites act as principal antioxidants by scavenging free radicals and stabilizing reactive oxygen species (ROS) [91–93]. Moreover, the tissue-specific effects, with flowers exhibiting higher antioxidant activity than leaves under certain light spectra, likely reflect the preferential accumulation of bioactive compounds, together with differences in glandular trichome density between leaves and flowers, and the differential responsiveness of phenolic biosynthetic pathways to spectral quality [94–96]. The observed variations in antioxidant enzyme activity and phenolic accumulation may be associated with light-induced modulation of reactive oxygen species (ROS) signaling [97]. Blue light, perceived by cryptochromes and phototropins, is known to enhance chloroplast development and photosynthetic electron transport, which can alter ROS production dynamics [98]. Moderate ROS levels can act as signaling molecules, activating antioxidant defense pathways and phenylpropanoid metabolism. Therefore, the spectral-dependent differences in antioxidant capacity observed in this study may reflect coordinated regulation between ROS balance and secondary metabolite biosynthesis [67, 99]. DPPH and FRAP assays are governed by distinct reaction mechanisms and therefore capture different dimensions of antioxidant behavior. The DPPH assay predominantly reflects free-radical quenching capacity through hydrogen atom transfer (HAT) and single-electron transfer (SET) processes, whereas the FRAP assay quantifies reducing potential via electron transfer (ET)-mediated reduction of Fe^3^⁺ under acidic conditions. Consequently, divergence between DPPH and FRAP responses under different spectral regimes likely represents shifts in the redox profile and chemical nature of accumulated antioxidants rather than inconsistent antioxidant performance. These assays should therefore be interpreted as complementary indicators of redox capacity rather than interchangeable metrics [100, 101]. Collectively, the findings indicate that spectral quality modulates growth–metabolism trade-offs in *T. parthenium* primarily through photoreceptor-mediated carbon allocation and ROS-related signaling rather than through alterations in PSII photochemical efficiency.

## Conclusion

This study demonstrates that manipulation of light spectral composition is an effective approach for modulating growth, photosynthetic performance, and secondary metabolite accumulation in *Tanacetum parthenium* indicating its potential role in regulating parthenolide biosynthesis. Among the tested treatments, red and white light were associated with the highest parthenolide accumulation in flowers, whereas red light significantly increased parthenolide content in leaves. In contrast, the 50R:50B treatment promoted reproductive development, while the 50R:50W treatment favored vegetative biomass accumulation, underscoring the spectrum-dependent regulation of distinct growth processes. Blue light markedly increased chlorophyll a, total soluble carbohydrates, and antioxidant capacity, highlighting its important role in supporting photosynthetic capacity and metabolic activity. Chlorophyll fluorescence analysis further revealed that 100% red light tended to enhance PSII photochemical efficiency, whereas 100% white light supported higher overall photosynthetic performance, suggesting that both narrow-band and broad-spectrum light regimes contribute differently to photosynthetic functioning. These organ-specific, spectrum-dependent responses highlight the complexity of light-regulated physiological networks in *T. parthenium*, likely mediated through photoreceptor-driven signaling networks. While the present study did not investigate molecular mechanisms, the observed effects of red light on parthenolide accumulation suggest the involvement of red light–responsive signaling pathways, which warrants further investigation using transcriptomic and metabolomic approaches. Collectively, these findings provide a physiological foundation for designing spectrum-specific lighting regimes aimed at optimizing biomass production or secondary metabolite accumulation in *T. parthenium*. Despite the significant physiological responses observed, this study has several limitations. Only a single light intensity (100 μmol m⁻^2^ s⁻^1^) and one genotype were evaluated under controlled greenhouse conditions. In addition, light treatments were applied during a reverse photoperiod regime, and economic or productivity assessments were beyond the scope of this work. Therefore, extrapolation to large-scale production systems should be made cautiously. Therefore, further investigations integrating molecular approaches, multiple light intensities, and long-term experiments are required to strengthen mechanistic understanding and support practical application.

## Supplementary Information

Supplementary Material 1

## Data Availability

All data supporting the findings of this study are available within the paper and its Supplementary Information.
