# Supplementary material for: Exploring the impact of light spectra on growth: unveiling the role of LED light in modulating phytochemical responses of feverfew (Tanacetum parthenium L.)
Source: BMC Plant Biol. 2026 Jul 2;26:1632. doi: 10.1186/s12870-026-09411-y (PMC13602796; doi:10.1186/s12870-026-09411-y)
Supplement: Supplementary file 1 [file 12870_2026_9411_MOESM1_ESM.docx]

**Supplementary Data:**

**Fig. S1** Chromatogram of Parthenolide of sample R-F (Red-Flower).

**Fig. S2** Chromatogram of Parthenolide of sample B-F (Blue-Flower).

**Fig. S3** Chromatogram of Parthenolide of sample W-G (White -Flower).

**Fig. S4** Chromatogram of Parthenolide of sample 50R:50B-F (50Red:50Blue -Flower).

**Fig. S5** Chromatogram of Parthenolide of sample 50R:50W-F (50Red -50White -Flower).

**Fig. S6** Chromatogram of Parthenolide of sample C-F (Control-Flower).

**Fig.S7** Chromatogram of Parthenolide of sample R-L (Red- Leaf).

**Fig. S8** Chromatogram of Parthenolide of sample B-L (Blue- Leaf).

**Fig.S9** Chromatogram of Parthenolide of sample W-L (White - Leaf).

**Fig. S10** Chromatogram of Parthenolide of sample 50R:50B-L (50Red:50Blue - Leaf).

**Fig. S11** Chromatogram of Parthenolide of sample 50R:50W-L (50Red -50White - Leaf).

**Fig. S12** Chromatogram of Parthenolide of sample C -L (Control-Leaf).
